# Liquid Biopsy in NSCLC: An Investigation with Multiple Clinical Implications

**DOI:** 10.3390/ijms241310803

**Published:** 2023-06-28

**Authors:** Elisa Bertoli, Elisa De Carlo, Debora Basile, Diego Zara, Brigida Stanzione, Monica Schiappacassi, Alessandro Del Conte, Michele Spina, Alessandra Bearz

**Affiliations:** 1 Department of Medical Oncology, Centro di Riferimento Oncologico di Aviano (CRO), IRCCS, 33081 Aviano, Italy; diego.zara@cro.it (D.Z.); brigida.stanzione@cro.it (B.S.); alessandro.delconte@cro.it (A.D.C.); mspina@cro.it (M.S.); abearz@cro.it (A.B.); 2 Department of Medicine (DAME), University of Udine, 33100 Udine, Italy; 3 Department of Medical Oncology, San Giovanni Di Dio Hospital, 88900 Crotone, Italy; deborabasile1090@gmail.com; 4 Molecular Oncology Unit, (OMMPPT) Department of Translational Research, Centro di Riferimento Oncologico di Aviano (CRO), IRCCS, 33081 Aviano, Italy; mschiappacassi@cro.it

**Keywords:** liquid biopsy, NSCLC, minimal residual disease, screening, precision medicine, immunotherapy, target therapy

## Abstract

Tissue biopsy is essential for NSCLC diagnosis and treatment management. Over the past decades, liquid biopsy has proven to be a powerful tool in clinical oncology, isolating tumor-derived entities from the blood. Liquid biopsy permits several advantages over tissue biopsy: it is non-invasive, and it should provide a better view of tumor heterogeneity, gene alterations, and clonal evolution. Consequentially, liquid biopsy has gained attention as a cancer biomarker tool, with growing clinical applications in NSCLC. In the era of precision medicine based on molecular typing, non-invasive genotyping methods became increasingly important due to the great number of oncogene drivers and the small tissue specimen often available. In our work, we comprehensively reviewed established and emerging applications of liquid biopsy in NSCLC. We made an excursus on laboratory analysis methods and the applications of liquid biopsy either in early or metastatic NSCLC disease settings. We deeply reviewed current data and future perspectives regarding screening, minimal residual disease, micrometastasis detection, and their implication in adjuvant and neoadjuvant therapy management. Moreover, we reviewed liquid biopsy diagnostic utility in the absence of tissue biopsy and its role in monitoring treatment response and emerging resistance in metastatic NSCLC treated with target therapy and immuno-therapy.

## 1. Introduction

Non-small cell lung cancer (NSCLC) counts approximately for 84% of all lung cancers and is a leading cause of cancer deaths worldwide [1].

In recent years, NSCLC embodied the paradigm of precision medicine application to clinical practice.

A better understanding of disease biology and identification of oncogenic driver alterations have led to the development of personalized therapeutic decision-making in advanced NSCLC, specifically in adenocarcinoma. The identification of druggable oncogene biomarkers has defined specific molecular NSCLC subsets [2,3].

Current international guidelines, including the International Society for the Study of Lung Cancer (IASCL) and the European Society for Medical Oncology (ESMO), have recommended testing NSCLC patients for multiple oncogenic alterations [4,5]. Predictive biomarkers include anaplastic lymphoma kinase (*ALK*) fusion oncogene, ROS proto-oncogene-1 receptor tyrosine kinase (*ROS1*) gene fusions, epidermal growth factor receptor (*EGFR*) gene mutations, B-RAF proto-oncogene, serine/threonine kinase (*BRAF* V600E) point mutations, neurotrophin tyrosine kinase (*NTRK*) gene fusions, c-mesenchymal-epithelial transition factor (*c-MET*) exon 14 (*METex14*) skipping mutations, human epidermal growth factor receptor 2 (*ERBB2*), and rearranged during transfection (*RET*) rearrangements, along with programmed Death-Ligand-1 (PD-L1) [6].

As the number of druggable molecular biomarkers has grown more and more, current guidelines recommend a multigene next-generation sequencing (NGS) approach over sequential single-gene testing [7].

Molecular analysis is the most-performed method on tumor tissue biospecimen. However, there are some disadvantages related to invasive procedure, inaccessible tumor biopsy anatomic site, potential inadequate tissue samples, and limitations to capture tumor heterogeneity, especially when multiple analyses are necessary to monitor tumor progression and treatment response.

Considering the tissue biopsy’s inherent risks and discomfort, liquid biopsy represents an effective non-invasive alternative methodology, performing a comprehensive genotyping profiling with NGS analysis [8].

In driver mutation-negative metastatic NSCLC, the therapeutic approach relies on immune checkpoint inhibitors (ICIs) with or without chemotherapy [9]. The mechanism of ICI treatment is to potentially block the immune checkpoint system and restore tumor recognition by the host immune system. However, there is an increasing need for biomarkers for patient selection and predicting ICIs efficacy [10].

Liquid biopsy offers multiple potential advantages over tissue, allowing extensive sequencing in all cancer stages. Therefore, potential clinical applications in lung cancer management are emerging, including early diagnosis, identification of minimum residual disease (MRD), detection of predictive and prognostic markers, assessment of resistance mechanisms, and monitoring treatment response [6].

The term “liquid biopsy” refers to different biofluid-derived analytes analyses (urine, cerebral spinal fluid, ascites, and pleural fluid), most commonly obtained via blood sampling [11]. The liquid biopsy approach includes a variety of methodologies, although plasma-derived circulating tumor DNA (ctDNA) is the most extensively analyzed derivate [12,13].

**Figure 1:**
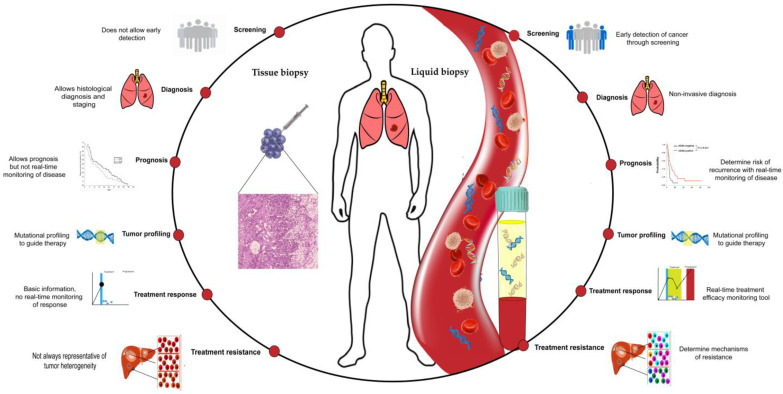
Liquid biopsy applications in comparison with tissue biopsy.

This review covers the evolving role and different applications of liquid biopsy in NSCLC patients, including screening, diagnosis, early-stage disease, and treatment of advanced disease (Figure 1).

## 2. Liquid Biopsy: In the Lab

Generating sensitive and specific technical approaches represents important challenges for the planned incorporation of non-invasive procedures, such as liquid biopsies. However, these approaches’ applicability and utility in routine decision-making are far from completely implemented.

**Figure 2:**
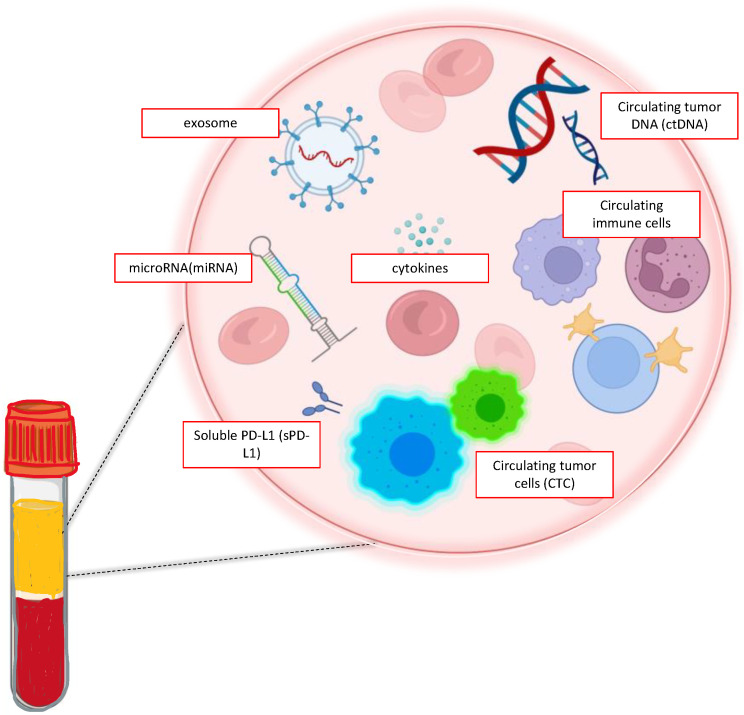
Liquid biopsy analytes.

The concept of liquid biopsies was born from the possibility to detect tumor biomarkers from a simple withdrawal of a biological fluid, usually blood. At present, circulating biomarkers typically detected by liquid biopsy are circulating tumor cells (CTCs), circulating cell-free DNA (cfDNA) and ctDNA, exosomes, microRNAs (miRNA), peripheral blood circulating RNA, and tumor-educated blood platelets (TEPs) [14]; CTCs, ctDNA, and exosomes are the most commonly detected biomarkers (see Figure 2).

### 2.1. CTCs

CTCs refer to tumor cells that are released from primary or metastatic tumors and can be identified in the peripheral blood, revealing relevant information regarding tumor heterogeneity and metastatic potential [15].

The ratio of CTCs to other cells in the blood is very low (1–10 CTC/mL blood in metastatic disease). Thus, a major issue concerning CTCs is the detection and isolation of CTCs from non-tumor cells in whole blood. The existing technologies used to isolate CTCs, not mutually exclusive and combinable, are essentially isolation based on immunoaffinity and biophysical properties [16,17].

The most common immunoaffinity-based CTC isolation method is based on antigen/biomarker expression on the CTC cell membrane. The gold-standard method is based on CellSearch system, the first immunoaffinity method approved by the Food and Drug Administration (FDA) in 2004, which captures CTCs that express common epithelial marker–epithelial cell adhesion molecule (EpCAM) and cytokeratins (CK 8, 18, and/or 19) [18]. It is worth noting that this principle cannot be applied to all CTC types. For example, CTCs affected by epithelial–mesenchymal transition and so negative for epithelial markers, are not detected by this method. NSCLC CTCs are often negative for EpCAM [18,19]. Moreover, CellSearch frequently captures dead cells.

The CTC detection based on physical features helps to isolate CTCs by cell size and electrical impedance. However, low recovery rates and manual processes have not yet been overcome [19,20,21]. The obstruction of mechanical microfiltres and microfluidic systems, as well as the peripheral blood cell adhesion to the surface filter, is the major impediment of these methods [21].

Thus, the standardization and clinical implementation of the developed technologies for CTC isolation remain a challenging issue for translational research and clinical practice. Despite these concerns, CTCs could represent the future an important tool to better characterize cancer heterogeneity, tumor biology, and cancer relapse.

### 2.2. ctDNA

Cell-free DNA (cfDNA) is a double-stranded fragmented DNA present in the bloodstream resulting from necrosis and apoptose mechanisms. ctDNA is the proportion of cfDNA of tumor cell derivation that encloses tumor-specific point mutations, rearrangements, and epigenetic features [22,23]. In the last years, major improvements have been conducted in ctDNA analysis and this approach appears readily available for routine clinical application [24,25]. The first technical approaches used were based on Droplet Digital PCR (ddPCR), a technique that allows the sensitive detection of specific mutations in specific sites of a given gene even present at low frequencies. With the development of ultra-deep NGS, amplicon-based approaches were successfully incorporated in routine labs [26], allowing for a broader assessment of the tumor–molecular profiling. Indeed, ctDNA was initially used for the detection of driver and secondary-resistance mutations during TKIs therapy.

Historically, the challenge of ctDNA was the limit of detection of sequencing technologies, as ctDNA counts for <1% of the total amount of circulating cfDNA. The development of ultra-deep NGS, detecting low-allele-frequency mutations with high coverage and appropriate bioinformatic methods discriminating mutations at low frequency from background noise strongly contributed to the global implementation of this approach as a diagnostic/theranostic tool [27,28,29]. Actually, many recent studies describe how high-resolution ctDNA testing may predict survival in metastatic NSCLC patients and can be integrated into large clinical trials [30,31]. Another peculiar characteristic of ctDNA is it has a short half-life [32], making it a potential dynamic biomarker of treatment response.

### 2.3. Exosomes and miRNA

Exosomes, with a size between 40–160 nm, are small extracellular vesicles that are released by most cells and play an important role in intercell communication [33].

Originating from the endosome system, exosomes transfer intercellular information carrying a variety of molecules, including proteins, lipids, nucleic acids (DNA, microRNA, mRNA), and other important information from the cell [34]. Various studies suggest that exosomes could be a novel biomarker in liquid biopsy because they are found to exist in almost all body fluids. However, the clinical application has been limited by the lack of elevated standard methods to analyze and separate components.

MicroRNAs (miRNA) are non-coding RNA with a size of about 18–25 nucleotides with regulatory functions; miRNAs especially regulate the expression of various oncogenes and tumor-suppressor genes, playing a key role in the pathological process of tumor development [35,36].

Therefore, altered levels of peripheral blood-circulating miRNA are associated with cancer development. Comprehensive studies have demonstrated miRNA in exosomes as a potential non-invasive biomarker for cancer-risk stratification and outcome prediction [37,38,39].

Quantitative real-time PCR (qRT-PCR) represents the gold standard technique for miRNA analysis. However, some disadvantages such as non-absolute quantification, false positives, and expensive equipment limit its clinical application.

### 2.4. TEPs

Tumor-educated platelets are defined as functional platelets with a distinct tumor-driven phenotype due to the transfer of tumor-related molecules from cancer cells to platelets [40]. However, it is not yet understood how platelets become educated and acquire distinct RNA and protein profiles. Most of the performed studies were focused on TEPs RNA content, demonstrating that spliced TEP–RNA surrogate signatures can provide specific information on cancer cell presence and promote tumor development [41,42].

## 3. Liquid Biopsy Diagnostic’s Application When the Tissue Is Unavailable

Tissue biopsy is as fundamental and challenging as in NSCLC. The principal limitation is the technical difficulty of reaching the biopsy site. Moreover, the NSCLC patient’s performance status (PS) may be so poor so as to contraindicate this invasive procedure. Another element to consider is the tumor heterogeneity; as a result, the biopsy could not be representative of the entire cancer cell population [43,44]. Finally, the material is often insufficient or inadequate for diagnosis, considerably complicating patient’s diagnostic process and delaying treatment. In this context, liquid biopsy can be a lifeline by allowing access to otherwise inaccessible information.

Multiple studies and meta-analyses evaluated liquid biopsy utility when the tumor tissue is missing, particularly for *EGFR* mutations detection. Globally, liquid biopsy has good specificity for the *EGFR* test on plasma, generally greater than 90%, but a lower sensitivity, varying between 50% and 80% depending on the technology employed [45,46]. It means that a positive result may allow target therapy. On the other hand, a negative result could be a false negative, significantly impacting the patient’s prognosis. Therefore, in these cases, it is necessary to perform a tissue biopsy, which represents the gold standard in the patient’s diagnostic process, especially when there are elements such as young age, no smoking history, and female sex, which strongly indicate the possibility of target mutation. [4]. All main international scientific societies recommend plasma genotyping in the event that sufficient tissue is not available, both at initial diagnosis as well as at disease progression [47]. In September 2014, the European Medicines Agency (EMA) advised the use of plasma ctDNA genotyping for patients with unavailable tissue sample. Moreover, the cobas^®^ *EGFR* Mutation Test v2 was approved by the US FDA to detect *EGFR* mutations (exon 19 deletion or exon 21 [L858R] substitution) in patients’ blood with the aim to start treatment with erlotinib, as well as to detect patients with T790M resistant mutation eligible for Osimertinib [48].

Different studies have demonstrated that in ctDNA *EGFR* mutant patients treated with EGFR TKIs, objective response rate (ORR), progression-free survival (PFS), and overall survival (OS) are similar to that in patients with EGFR mutations detected in tumor tissue [49,50,51,52]. Moreover, mutation status concordance between 652 matched tumor and plasma samples was 94.3%, with a sensitivity of 65.7% and a specificity of 99.8% [52]. A systematic review and meta-analysis assessed the diagnostic performance of cfDNA, compared with tissues, confirming that the detection of *EGFR* mutation by cfDNA is of adequate diagnostic accuracy, (pooled sensitivity of 0.674 (95% CI: 0.517–0.800) and a specificity of 0.935 (95% CI: 0.888–0.963) [45]. Similar results were reported by Qiu et al. [46]. However, all patients in the above studies had clear pathological diagnoses from tissue biopsy.

Deng et al. conducted a small prospective single–center study to explore the role of ctDNA in detecting *EGFR* mutations in the plasma of patients with suspected advanced NSCLC but without a pathologic diagnosis and to evaluate the efficacy of first-generation EGFR TKIs in the first-line setting [53]. After a median follow-up of 12 months, median PFS was 10 months and median OS was not reached. Similar results have been described in a Phase 2 study with Icotinib [54], confirming the possibility to rely on liquid biopsy to start treatment with EGFR TKIs, even if a pathological diagnosis is unavailable.

While for *EGFR*-mutated NSCLC, there are consolidated data, the role of liquid biopsy in detecting other mutations has been evaluated more thoroughly only in recent years, thanks to the introduction of NGS [6]. Raez et al. demonstrated that concordance between liquid and tissue NGS ranged between 94.8% and 100%, not only for the detection of *EGFR* but also for the principal actionable mutations (*BRAF* (8%) *ALK* (6%), *MET* (6%) *NTRK* (2%), and *ROS1* (2%)) [55]. In a wide study published by Mack et al., clinical impact of ctDNA in NSCLC was evaluated in over 8000 cases. Driver oncogene mutations (*EGFR*, (26.4%), *MET* (6.1%), *BRAF* (2.8%)) and *ALK*, *RET*, and *ROS1* fusions (2.3%) were detected in 48% of patients [56]. Although extrapolated from a subgroup analysis, the interesting data is that the ctDNA analysis allowed for the detection of driver mutations by 65% over tissue-based testing at diagnosis, and that the responses to target therapy based on ctDNA were comparable to those reported from tissue analysis [56]. Comparable data have been published by Zugazagoitia et al. [57].

The BFAST trial is an ongoing six-cohort study prospectively evaluating the relationship between blood-based biomarkers (*ALK*, *RET*, *ROS1*, *BRAF*, *EGFR exon 20*, blood tumor mutational burden (bTMB)) and clinical activity of target or immune-therapy in first-line NSCLC setting patients who only underwent NGS for the detection of actionable genetic alterations [58]. Preliminary results from the *ALK* cohort receiving alectinib have been reported. After a median follow-up of 12.6 months, ORR was 87.4% (95% confidence interval [CI]: 78.5–93.5), intracranial ORR 91.4% (95% CI: 76.9–98.2), 12-month duration of response (DOR) 75.9% (95% CI: 63.6–88.2) with a median PFS not reached. These results confirm the feasibility of plasma NGS analysis for detection of *ALK* fusion [58].

In a single-arm Phase II study, patients with advanced NSCLC and *METex14* mutation identified by liquid biopsy or tumor biopsy were candidates to receive tepotinib [59]. At data cut-off, ORR was 51.4% and 41.5%, respectively. Although preliminary, these data show a promising activity for tepotinib whether the mutation is detected with tissue biopsy or with liquid biopsy.

All data considered, the evaluation of the mutational status on liquid biopsy with NGS is currently desirable as a possible alternative in newly diagnosed advanced NSCLC patients when the quantity and/or quality of available tissue is not adequate.

## 4. Liquid Biopsy in Screening and in Early Nsclc Disease Stage

### 4.1. Screening

Lung cancer is an important and complicated problem of public health. In recent years, the use of low-dose computed tomography (CT) was widespread in the screening phase. Low-dose CT screening compared to chest radiography was demonstrated in the National Lung Screening Trial to reduce lung cancer-related and overall mortality in selected patients [60]. Despite the advantage, screening with the use of low-dose CT had some limitation such as overdiagnosis, false positive, and the potential risk of radiation exposure; the use of biomarker could be useful to overcome the low dose CT limitation. Several kinds of biomarkers were evaluated in screening and early detection of lung cancer.

A large amount of evidence reported the ability of several promising blood-biomarkers to distinguish between NSCLC and healthy samples. These data suggest that the clinical integration of liquid biopsy into the screening programs remains an important achievement to be pursued to improve lung cancer screening algorithms and their implementation in clinical practice.

In a case-control study, Sozzi et al. firstly determined a higher level of plasma DNA in patients with lung cancer compared with control subjects [61]. Based on this evidence, the same group determined plasma DNA levels in 1035 heavy smokers monitored by annual CT for 5 years. No differences in baseline plasma DNA concentration between individuals who developed CT-detected lung cancers and cancer-free control subjects were determined, even if DNA levels significantly increased as the time from lung cancer diagnosis decreased, with a possible role of plasma DNA level in early cancer screening [62]. In the following years, many other studies reported the ability of ctDNA to distinguish between NSCLC-affected patients and healthy subjects [63,64,65,66]. Available data indicate that ctDNA concentration may be a valuable tool to be used for lung cancer screening and early detection.

Another interesting approach is the use of cfDNA methylation analysis [67,68]. Of particular interest is the multi-cancer early detection (MCED) test that uses the methylation patterns of cell-free DNA to detect a shared cancer signal from more than 50 different cancer types. The test demonstrated a specificity of 99.5%, an overall sensitivity across cancer classes of 51.5%, and an overall accuracy of cancer signal origin prediction of 88.7% in true positives. In NSCLC, sensitivity was 51.5%, 16.8%, 40.4%, 77%, and 90.1% in overall, Stage I, II, III, and IV, respectively [69]. Moreover, Hubbell et al. suggested that the MCED test could reduce late-stage (III + IV) incidence by 78% and prevent 26% of all cancer-related deaths in persons aged 50–79 [70]. The NHS–Galleri trial (ISRCTN91431511), a randomized controlled trial that aims to establish whether the screening with MCED test of asymptomatic individuals can reduce late-stage cancer incidence [71], is ongoing.

Several studies compared the expression of miRNA in lung cancer and healthy tissue to define miRNA panels that could help in early detection and screening of lung cancer [72,73,74]. Some examples of particular interest are plasma microRNA signature classifier (MSC) and miR-Test. MSC, a signature formed by 24 miRNAs, was retrospectively evaluated in plasma samples collected within the Multicenter Italian Lung Detection (MILD) trial. MSC showed a significant diagnostic performance for lung cancer detection with a sensitivity of 87% and a specificity of 81%. Notably, the addiction of MSC tests for low-dose chest CT scan (LDCT) would raise screening sensitivity from 84% for LDCT alone to 98% [75]. Based on these results, the BioMILD study was designed to assess the value of the blood MSC assay at the time of baseline LDCT with the goal of personalizing lung cancer screening. The study demonstrated that MSC+ participants had a two-fold higher lung cancer incidence than MSC− participants, and that there was no evidence that the MSC effect differed between CT+ and CT− subjects. Notably, the incidence of lung cancer was particularly high in CT+/MSC+ participants [76]. Similarly, the miR-Test, a blood test based on serum miRNAs in high-risk individuals, presented an overall accuracy, sensitivity, and specificity of 74.9%, 77.8%, and 74.8%, respectively. Particularly relevant is that no major differences were observed in the sensitivity of the detection of different tumor stages: 69% in Stage I, and 71.9% in Stages II to III [77]. A systematic review and metanalysis that included a total of 134 studies (6919 patients with lung cancer and 7064 controls) based on miRNA evaluation highlighted that circulating miRNAs had a good diagnostic performance in lung cancers, with a sensitivity of 83% and a specificity of 84%. Notably, at a subgroup analysis, the combinations of miRNAs were more complete indicators than individual miRNAs. In particular, a panel of miRNAs (miR-21-5p, miR-223-3p, miR-155-5p and miR-126-3p) showed a potential biomarker activity [78]. All of this evidence suggests a potential capacity of some miRNA panels to discern NSCLC from benign nodules.

Interestingly, miRNA has also been evaluated in sputum with good sensitivity and specificity in an approach that could improve CT scan specificity for the diagnosis of lung cancer in smokers [79,80].

Finally, miRNAs could also be detected in exosomes, and significantly higher exosomal miRNA have been detected in lung adenocarcinoma compared to the control. These data suggest that circulating exosomal miRNA might be useful as a screening test for lung adenocarcinoma. Moreover, different panels of microRNAs derived from circulating exosomes were studied both in screening and in diagnostic setting. In screening settings, four microRNAs (miR-378a, miR-379, miR-139-5p, and miR-200b-5p) showed the ability to divide the population between nodule (lung adenocarcinomas and carcinomas) and non-nodule (healthy former smokers) subjects with 97.5% sensitivity, 72% specificity, and an AUC–ROC of 90.8%. In diagnostic settings, six microRNAs (miR-151a-5p, miR-30a-3p, miR-200b-5p, miR-629, miR-100, and miR-154-3p) demonstrated the capacity to discriminate between lung adenocarcinoma and granuloma, and the test had a sensitivity of 96%, as well as a specificity of 60% [81,82].

Different types of CTCs showed a sensitivity and specificity in the diagnosis of NSCLC. For example, folate receptor-positive CTCs showed the highest diagnostic efficiency compared with the existing clinical biomarkers [83]. Similar results were reported with a morphological classification of circulating cells [84]. Intriguingly, Ilie et al. examined the presence of CTCs with complementing CT scans in chronic obstructive pulmonary disease (COPD) patients without clinically detectable lung cancer, as well as in subjects without COPD. CTCs were detected in 3% of COPD patients; conversely, no CTCs were detected in healthy individuals. The CTC-positive COPD patients underwent annual surveillance by CT scan screening, which detected lung nodules. This led to prompt surgical resection and histopathological diagnosis of early-stage lung cancer (pT1aN0M0 in all cases). These data suggested a possible role of CTCs in the protocol screening for “at-risk” patients [85].

Other applications of liquid biopsy explored are the identification of TEPs with associated marker genes as a predictive factor of the existence of cancers [86], the development of a machine-learning method (e.g., Lung-CLiP [87]), and the constitution of an Olink customized panel (INTEGRAL panel) based on PEA technology that can measure up to 21 relevant proteins [88]. An ongoing study evaluates this panel in pre-low-dose CT to identify people likely to benefit from screening, and in post-low-dose CT to differentiate benign versus malignant nodules [88].

### 4.2. Neoadjuvant Setting

A promising application of liquid biopsy in NSCLC is the assessment of patients undergoing neoadjuvant therapy. Neoadjuvant chemotherapy resulted in a decrease of methylation in cfDNA circulating in the blood, which became more pronounced in the post-tumor resection period. Importantly, a methylated allele concentration increase was detected in patients manifesting disease relapse [89].

Following, the utility of ctDNA was evaluated in some trial of neoadjuvant immunotherapy.

In the NADIM trial, both low pre-treatment levels of ctDNA and undetectable ctDNA levels after neoadjuvant chemo-immunotherapy treatment were significantly associated with PSF and OS. Moreover, undetectable ctDNA levels after neoadjuvant treatment outperformed radiologic responses in the prediction of OS [90]. In addition, the results of CheckMate816 support the hypothesis that clearance during neoadjuvant chemo-immunotherapy may be an early predictor of favourable outcomes. Particularly, event-free survival and percentage of patients with a pathological complete response appeared higher in patients with ctDNA clearance than in those without ctDNA clearance in both treatment groups [91]. Similarly, the ctDNA dynamics evaluation in the LCMC3 study confirmed that ctDNA level reductions post-neoadjuvant therapy correlated with pathologic and radiologic responses in patients treated with atezolizumab. Furthermore, the absence of ctDNA post-surgery correlated with a better 2-year disease-free survival (DFS) [92]. Moreover, the biomarker analyses showed that pre-treatment peripheral blood immune cell profiles may predict major pathological response in atezolizumab-treated patients with respectable NSCLC [93].

Yue et al., in a retrospective study that enrolled Stage IB–IIIA NSCLC treated with neoadjuvant immunotherapy combined with chemotherapy, dual immunotherapy, or chemotherapy alone, confirmed the concordance of ctDNA change during neoadjuvant therapy with the pathologic response. The authors also reported the prognostic value of perioperative ctDNA in predicting recurrence [94]. These data have been analyzed in a meta-analysis: a higher percentage of patients with a major pathological response or pathological complete response (pCR), among those with a reduction in ctDNA concentration (33%–86%) than among those without ctDNA decrease (0–17%), was reported with a strong correlation between a pathological response and a reduction of ctDNA concentration (*p* < 0.00001). In addition, significantly improved long-term survivals were both observed for patients with pCR and ctDNA clearance after neoadjuvant therapy [95].

The results of other randomized trials with neoadjuvant immunotherapy-exclusive regimens, or in combination with chemotherapy, may provide more rigorous evidence if and how liquid biopsy can be utilized optimally for earlier NSCLC treatment.

### 4.3. Minimal Residual Disease (MRD) and Adjuvant Setting

The use of liquid biopsy in early-stage NSCLC represents a partially unexplored research field in which various experimental studies have proliferated in recent years.

Liquid biopsy implementation in clinical practice to assess the presence of MRD is an important translational research field. MRD refers to the detection of any tumor-derived material in the blood after curative-intent treatment, earlier than standard-of-care radiologic imaging [15]. To note, the assessment of MRD remains challenging. ctDNA is one of the most advanced and frequently investigated technologies to detect MRD.

In Stages I–III, with NSCLC patients who underwent curative-intent treatment, recurrence represents a challenge. The 5-year survival rate declines from 90% for Stage IA1 to 41% for Stage IIIA in resected lung cancer patients [96]. The Lung Adjuvant Cisplatin Evaluation (LACE) analysis demonstrated a 5-year survival benefit of 5.4% from adjuvant chemotherapy, with an overall hazard ratio (HR) of death of 0.89 (95% CI, 0.82 to 0.96; *p* = 0.005) [97]. Durvalumab administration after chemo-radiotherapy (CRT) has improved the outcome in unresectable Stage III NSCLC patients [98].

After radical surgery or CRT, MRD detection could allow medical oncologists to identify patients at the highest risk for recurrence; they benefit most from adjuvant therapy. In this scenario, ctDNA has emerged as an independent predictive marker of relapse in early-stage NSCLC. Several studies have demonstrated the ctDNA sensitivity in MRD detection. These studies have investigated the dynamic changes of ctDNA in early-stage NSCLC patients, proving a rapid decline in ctDNA levels after surgical resection [26,99,100,101]. Conversely, detectable plasma ctDNA after resection correlates with residual/recurrent disease.

Undoubtedly, new technology development allowed for the broader use of liquid biopsy in early NSCLC. Cancer Personalized Profiling by deep sequencing (CAPP-Seq) is a highly sensitive NGS-based method that is able to quantify ctDNA and detect multiple classes of somatic mutations per patient, achieving lower detection limits of about 0.02% without requiring a personalized assays creation [65,102]. ctDNA levels significantly correlated with tumor volume and could evaluate treatment response and residual disease. In the TRACERx trial, Abbosh et al. evaluated ctDNA to detect and profile residual tumor cells persisting after curative intent therapy, demonstrating that patients with undetectable presurgical ctDNA have better outcomes. Moreover, the subclonal architecture of low ctDNA concentrations was studied through bioinformatics, revealing that patients with polyclonal metastatic dissemination have poor clinical outcomes [30].

A cornerstone of studies on liquid biopsy in early NSCLC was published by Chadhuri et al. [103]. To identify MRD in early-stage lung cancer patients, CAPP-Seq was retrospectively used to search for ctDNA in 255 blood and tissue samples from 40 Stage I–III patients who underwent curative-intent treatment, as well as 54 healthy adults. ctDNA has been analyzed before treatment, and the subsequent follow-up visits concomitant with radiological imaging. Pre-treatment ctDNA concentration highly correlated with metabolic tumor volume and stage. All patients with detectable ctDNA in at least one post-treatment evaluation experienced recurrence. Intriguingly, ctDNA was detected in the first post-treatment blood sample in 94% of patients with relapsed disease, suggesting that ctDNA is a sensible tool for detecting MRD. Furthermore, the trial has demonstrated that ctDNA is capable of anticipating imaging-documented progression of a median of 5.2 months in 72% of patients. Interestingly, freedom from progression (FFP), disease-specific survival, and OS were significantly lower in patients with detectable ctDNA at any post-treatment time point compared to those with undetectable ctDNA after curative-intent therapy.

Therefore, the results of this trial suggest the potential clinical application of ctDNA in the identification of patients who significantly benefit from adjuvant therapy, avoiding overtreatment and futile toxicities.

To evaluate liquid biopsy in the setting of locally advanced NSCLC and the impact of MRD on consolidation immunotherapy, Moding et al. enrolled patients with unresectable Stages IIB–IIIB NSCLC who underwent CRT therapy with or without ICI consolidation [104]. Using CAPP-Seq, they determined that patients with undetectable ctDNA after CRT demonstrated better outcomes regardless of the administration of immunotherapy. In contrast, patients with MRD detection after CRT and the reception of ICIs had significantly better survival compared with patients who did not receive ICIs.

A recent meta-analysis and systematic review evaluated the diagnostic and prognostic value of liquid biopsy for early-stage NSCLC, involving studies on the classical serum biomarkers, CTCs, ctDNA, methylation signatures, and microRNAs [95]. Thirty-four studies were analyzed for diagnostic assessment. The analysis suggests that the biomarkers demonstrated a similar and acceptable effectiveness for the early detection of lung cancer, with the area under the curve (AUCs) ranging from 0.84 to 0.87. A lower diagnostic accuracy correlated with Stage I.

To evaluate the liquid biopsy prognostic value, 21 studies were analyzed, including 2143 patients. Shen et al. demonstrated that MRD blood presence was a strong predictor of disease relapse (recurrence-free survival (RFS), HR, 4.95; 95% CI, 3.06–8.02; *p* < 0.001) and shorter OS (HR, 3.93; 95% CI, 1.97–7.83; *p* < 0.001), with an average lead time of 179 ± 74 days between molecular recurrence and radiographic progression. Moreover, biomarkers positive both in the presurgical and post-chemotherapy blood samples were associated with significantly inferior RFS and OS. Interestingly, adjuvant therapy was associated with a significant RFS benefit in patients with ctDNA-based MRD (HR, 0.27; 95% CI, 0.17–0.44; *p* < 0.001), while the survival benefit was not found in patients with undetectable MRD (HR, 1.51; 95% CI, 0.81–2.79; *p* = 0.19) [95].

In Table 1, we demonstrate resumed ctDNA performances to detect MRD in early-stage NSCLC.

Increasing evidence suggests that liquid biopsy represents a valuable tool for ctDNA-based MRD detection and could be a prognostic biomarker in early-stage NSCLC. Therefore, prospective trials will be required to validate this approach and to evaluate if adjuvant personalized treatment based on MRD detection will improve NSCLC survival.

## 5. Liquid Biopsy in Oncogene Addicted Advanced Nsclc: A Focus on Target Therapies

Mounting evidence has detailed the importance of the underlined molecular biology and cancer heterogeneity as critical factors for cancer prognosis and a response to anticancer treatment [105,106].

Tissue biopsy still represents the gold standard for lung cancer diagnosis and treatment. However, it is a static procedure that is not capable of detecting molecular heterogeneity and secondary resistances prompted by treatments. For example, it is proven that ctDNA analysis is more sensible in detecting KRAS mutation than tissue analysis [107]. Moreover, the failure rate for an adequate molecular profiling is approximately 30% of cases [108].

For patients with a new diagnosis of NSCLC, the initial genotyping with liquid biopsy could be applied for several uses: (i) In the case of available tumor tissues, when the amount of tissue is so low that it is not possible to perform all the molecular analyses; (ii) In the case of tissue biopsy that is not executable or not available; (iii) To detect relevant targetable oncogenic alterations [6]. Furthermore, liquid biopsy during anticancer therapy is useful to monitor disease progression, particularly to detect treatment response and resistance.

Over the last years, liquid biopsy is widely used in oncogene-driven advanced and metastatic NSCLC to assess *EGFR* mutational status, either in first-line patients or after acquired resistance to EGFR TKIs (see Table 2 for details) [49,52,109,110,111,112,113].

Many studies evaluated the prognostic role of ctDNA in NSCLC patients harboring EGFR mutations, demonstrating shorter survival outcomes. A recent analysis demonstrated that EGFR mutation detected with ctDNA has been associated with higher frequency of metastatization and reduced DFS. Accordingly, Liu et al. reported, in the EGFR-mutated population, that allele frequency heterogeneity assessed using ctDNA was significantly associated with poorer OS [116]. Moreover, NSCLC patients with EGFR mutation detected on ctDNA and, particularly, with the ctDNA copy number alteration showed a shorter progression-free survival (PFS) and OS [117]. Of note, L858R mutation is associated with poorer OS (13.7 months) compared to wild-type tumors (27.7 months) [118]. TP53 mutation discovered in EGFR mutant tumors, especially on Exons 6 and 7, assessed on ctDNA was also associated with worse PFS and OS [119].

Interestingly, Nygaard et al. reported a significantly poorer PFS (3 vs. 5.6 months) and OS (4.8 vs. 9.5 months) in patients with advanced NSCLC with KRAS mutation detected on ctDNA [120]. Several studies observed an inferior survival outcomes according to ctDNA levels, as well as the association between ctDNA levels and metastatic spreading [121].

ALK-positive tumors were also extensively studied. ctDNA levels were associated with disease burden. Furthermore, ALK-positive ctDNA levels were also associated with a probability of recurrence [122]. Notably, ctDNA clearance during treatment with *ALK* inhibitors was also established as a marker of better prognosis [123]. Similarly, BRAF mutation from ctDNA was also associated with reduced survival [124].

First- and second-generation TKIs have been recognized regarding the treatment of choice for many years for *EGFR* mutant tumors. However, the onset of acquired resistance often occurs over time. Hence, the detection of T790M mutation in Exon 20, which represents approximately 50% of resistance mechanisms, allowed for the commencement of EGFR TKIs of the third generation. Nonetheless, other off-target resistance mechanisms could occur during treatment with EGFR inhibitors, including *MET* amplification (approximately 5–20% of patients), *ERBB2* amplification, activation of *AXL* pathway, or mutations of *BRAF* and *PI3KCA* [125,126,127,128].

In the AURA 3 study, a translational analysis testing ctDNA genomic profiles in patients who progressed to second-line osimertinib discovered *MET* amplification in 19% of patients and *EGFR C797S* in 15% of cases. Moreover, 49% of patients who progressed during osimertinib lost T790M mutation owing to *ERBB2* amplification (5%), *PI3KCA* or *BRAF* mutations (4% and 4%, respectively), *KRAS* mutations (1%), SCLC transformation, and gene fusion [129]. An exploratory study conducted through BEAMing technology on the Phase I AURA identified that L858R mutation and Exon 19 deletion with a sensitivity of 85% and 82%, respectively, confirmed the predictive role of T790M mutation [114]. The latter’s sensitivity can vary according the technology used, probably due to the tumor heterogeneity and burden [112,113,115,130]. Thus, liquid biopsy allows for the tracking of these biological behaviors. EGFR T790M/activating mutation ratio and *EGFR* allele frequency could provide additional information on response during osimertinib treatment [131,132]. International guidelines have established the use of liquid biopsy for patients progressing to TKI [6].

Notably, the treatment of the first line has been changed in the last year based on the results obtained from the FLAURA Phase III trial, which established osimertinib as a first-line treatment for *EGFR* mutant NSCLC, namely introducing new challenges for liquid biopsy applications. The prognostic implication of liquid biopsy has been assessed in that trial; base-line allele frequencies of the *EGFR* mutant was associated with survival outcomes. A better PFS was reported in patients without detectable *EGFR* mutation for both arms (osimertinib 23.5 months and first-generation TKIs 15 months) compared to those with positive mutations (osimertinib 15.2 and first-generation TKI 9.7 months). Furthermore, the clearance of mutant *EGFR* after TKIs was associated with improved PFS and OS [133,134]. The main acquired resistance to osimertinib administered in this setting is *MET* amplification, detected in 15–20% of patients examined with NGS ctDNA test, as reported also by the ELIOS study [135]. In fact, in the FLAURA study, 91 patients tested with liquid biopsy. They then progressed to first-line osimertinib, acquired *MET* amplification (15%), *ERBB2* amplification, and mutation of *EGFR C797S*, *PIK3CA,* and *RAS* [136]. However, acquired resistance mechanism to first-line osimertinib is not as explored as those developed in the second line. Plasma NGS could identify both on-target and off-target acquired alterations, providing a real-time evaluation of the wide cancer molecular profile and clonal evolution. Thus, it provides the opportunity for more appropriate treatment sequences to match to the molecular status.

No target therapies are available after the acquisition of mutation during osimertinib treatment. Anyway, different trials targeting *MET* amplifications are currently ongoing: amivantamab plus lazertinib (NCT04077463, Chrysalis-2), tepotinib plus osimertinib (INSIGHT-2, NCT03940703), and osimertininib plus savolitinb (TATTON, NCT02143466). Translational analyses evaluating tissue biopsy and plasma collection are planned in trials to validate the use of liquid biopsy to identify MET amplification. Meanwhile, preliminary results by the NCT03178552 trial reported the feasibility of plasma NGS for the identification of *ALK* fusion in patients treated with alectinib. Likewise, the NCT02864992 revealed that liquid biopsy is a promising tool for MET amplification [59,137,138,139].

In recent years, several additional actionable oncogene drivers, such as mutations in *BRAF*, *ALK*, and *KRAS* mutation, and gene fusions involving *ALK*, *ROS1*, *NTRK1*, *NTRK2*, *NTRK3*, and *RET* could be targeted by drugs available for clinical use. Further, *ERBB2* mutations, *NRG1* fusions, *EGFR exon 20* insertions, *NTRK* rearrangements, and tumor molecular burden (TMB) could be promising targets in lung cancer and could be implemented in the test panels [1].

ESMO guidelines currently recommend the assessment of oncogene drivers and PD–L1 expression levels in advanced NSCLC [4,5]. In the future, plasma collection with a broad NGS analysis after progression could be able to detect additional druggable targets, particularly, *ALK* and *RET* rearrangements and *BRAF* mutations [140,141]. The BFAST Phase I/II trial showed the predictive role of liquid biopsy performed with NGS analysis in detecting *ALK* rearrangements [142]. Likewise, the eXalt2, a Phase I/II study, reported a concordance rate of 91% between tumor tissue and liquid biopsy in patients with *ALK* rearrangements [143]. A key issue, in the future, is the evaluation of acquired alterations during treatment with ALK inhibitors. The NCT03737994 study collects tissue biopsy and plasma to detect secondary resistance mechanisms during treatment with second-generation ALK inhibitors. Interestingly, treatment after progression on second-generation ALK TKIs is biomarker-driven. An important aspect to underline is that, in case of solely encephalic disease progression, the liquid biopsy on blood may not be informative and should be instead done on cerebrospinal fluid to detect the possible presence of resistance mechanisms [144].

To note, there is little data regarding the analysis of gene fusions on cell-free RNA and DNA-based NGS fails, for example, to capture NTRK fusions. Thus, tissue biopsy remains the gold standard for gene-fusion detection [145].

## 6. Liquid Biopsy in Non-Oncogene Addicted Advanced Nsclc: A Focus on Immunotherapy

Alongside chemotherapy, ICIs are the mainstay of non-oncogene-addicted NSCLC disease treatment. The decision between first-line immunotherapy alone or in combination with chemotherapy is based on the levels of expression of PD-L1 on the cell surface evaluated by immunohistochemistry on tumor tissues [5,146]. Immunotherapy in second-line monotherapy or in first-line as monotherapy in patients with PD-L1 > 50% and in combination with front-line chemotherapy in patients with PD-L1 < 50% changed the history of patients with advanced non-NSCLC non-oncogene addicted with 5-year OS of 20% in non-selected patients, and up to 50% in patients with high PD-L1 expression [5,147]. Although the benefit from ICI is higher in patients with high PD-L1 levels, patients with PD-L1 < 1% also benefit from immunotherapy. Moreover, a proportion of patients with high levels of PD-L1 do not respond to the treatment of ICIs [10,148]. Therefore, the evaluation of PD-L1 in immunohistochemistry is not a completely satisfying biomarker for predicting the response to ICIs [149]. Indeed, also considering the toxicity of these treatments, searching for new biomarkers is crucial to selecting patients that will benefit from immunotherapy more accurately, limiting ineffective therapies that may lead to immune-related adverse events.

In recent years, liquid biopsy has been explored in this field to search for new predictive biomarkers of response to ICIs, monitor the course of immunotherapy to detect patients who do not benefit from treatment early, and identify genetic alterations associated with a resistance to ICIs.

### 6.1. ICI Response or Resistance Prediction

Among biomarkers under investigation, TMB should be the most emphasized. It is defined as the number of somatic mutations per mega base of the sequenced genome. The FDA recently approved pembrolizumab for solid tumors with a high TMB (≥10 mutations/Mb) [150], although data supporting the predictive role of TMB tissue immunotherapy response in NSCLC are conflicting [151]. The evaluation of the TMB on blood, analyzing the ctDNA, was validated a few years ago in the BFAST trial [152] and overcame the obstacle of the scarcity of tumor tissue availability in NSCLC for TMB evaluation. To date, available data differ in associating baseline bTMB with clinical outcomes in patients with NSCLC treated with ICIs [153,154,155,156,157]. Basically, this can be related to the lack of standardization in the bTMB analysis methods, in the different cut-offs chosen to define a high bTMB, the mutation panel, and the gene sequences analyzed [158]. There are several ongoing clinical trials evaluating the predictive value of bTMB, including the aforementioned BFAST (NCT03178552). Results from NCT04636047, an ongoing prospective study evaluating NGS-based comprehensive genomic ctDNA panel, including bTMB, in NSCLC treated with ICIs are awaited.

In advanced NSCLC, the presence of CTCs has been associated with a poor prognosis [159,160]. In addition, in patients with ICIs who were treated with NSCLC, the presence of CTCs at the baseline was associated with the worst response and survival rate (see Table 2 for references and details). To note, the only FDA-approved method for CTC research is the CellSearch system [161].

The expression of PD-L1 was also evaluated on CTCs [162]. Interestingly, PDL1 was more frequently expressed in CTCs than in tissue samples in NSCLC, suggesting that CTCs may better capture tumor heterogeneity [163]. Although it has been defined as PD-L1, the evaluation of CTCs may be feasible and a useful tool in NSCLC, although its predictive role in immunotherapy response has not been uniquely demonstrated [163,164]. Among the ongoing studies evaluating the predictive role of CTC-PD-L1, there are: ALCINA trial (NCT02866149), Immunopredict (NCT02827344), and NCT04490564.

Moreover, the evaluation of PD-L1 expression on exosomes (exoPD-L1) has been investigated. If elevated basal exoPD-L1 levels correlate with a worse prognosis, an early increase in these levels is found in immunotherapy responders [165,166,167,168].

PD-L1 is also present in a soluble form (sPD-L1). sPD-L1 is supposed to maintain the PD-L1 characteristic immunosuppressive function. Despite conflicting data, the presence of high levels of pre-treatment sPD-L1 appears to be a negative predictive factor of response and an independent negative prognostic factor in patients with NSCLC treated with ICIs [169,170,171].

Although it has less impact than oncogene-addicted disease, the use of liquid biopsy was explored to identify mutations associated with resistance or sensitivity in ctDNA during ICI therapy. Namely, *PTEN* and *STK11* mutation, and *STK11*/*KRAS* and *KEAP1*/*KRAS* co-mutations, were found to predict poor prognosis, as well as whether *KRAS* and *TP53* transversion mutations could correlate with good response [172,173,174,175].

Both circulating miRNA and miRNA derived from exosomes were explored in patients with NSCLC treated with ICIs. The upregulation of specific exosomal miRNA and the downregulation of others has been correlated with the resistance or response to ICIs [176]. The same was also described for circulating miRNA [177,178], with which a signature was also created that would be able to identify a subgroup of patients who do not benefit from the ICIs [179]. However, it is important to underline that, to date, there is no consensus in the methodology of isolation and analysis of miRNA, and that all these studies are based on very small numbers.

### 6.2. Treatment Response Monitoring

ctDNA is probably the most studied and promising liquid biopsy biomarker. Shreds of evidence support the predictive role of clinical benefits in patients with NSCLC treated with ICIs, although its clinical relevance is still under investigation. The most intriguing results relate to the dynamic longitudinal monitoring of ctDNA during treatment. A reduction in ctDNA levels during ICI treatment significantly correlated with higher response rates and longer PFS and OS. More precisely, an early variation (3–12 weeks from ICIs start) in the allelic fraction of ctDNA correlates with the radiological response and clinical outcomes [180,181,182,183,184,185,186,187] (see Table 3 for other references and details).

One challenge of immuno-oncology is undoubtedly the interpretation of pseudo progression, early progression, and delayed radiological response [191]. Considering, as mentioned above, that the variation of the ctDNA is early and that a concordance between the reduction of the ctDNA and the radiological response has been proven [180,186], the fact that the clearance of ctDNA on liquid biopsy is detectable in advance of the radiological restaging makes this method a tool of enormous potential in the differential diagnosis between pseudo progression and actual progression, as well as between missed/early progression or only delayed radiological responses [180,182,192]. Despite these exciting data, the actual therapeutic impact on clinical decision-making has yet to be evaluated in clinical trials.

Another open question is the optimal duration of immunotherapy in responders. An interesting starting point, which needs further investigation, is the possibility to use the ctDNA to establish the risk of progression in long-responders, similarly to the concept of MRD described above [193].

Most evidence supports that an increase in CTC during ICIs is predictive of disease progression and non-response to treatment [157,194,195,196,197]. Despite the small numbers of patients of these studies and the different methods of detection of CTC, the monitoring of CTCs during ICIs begins to have solid data to support its possible future application in clinical practice.

## 7. Discussion and Conclusions

Liquid biopsy is a very powerful and minimally invasive approach for cancer management that provides important information through peripheral blood analysis.

Technological advances in DNA sequencing have improved the accurate detection of ctDNA, expanding its potential clinical applications.

In the diagnostic process, cyto-histopathologic evaluation of the tissue specimen remains the gold standard for cancer diagnosis and for PD-L1 testing.

Not yet considered a standard technique, ctDNA analysis has been explored in numerous clinical phases of lung cancer, from screening and early diagnosis to prognostic factor identification in initial disease to the molecular characterization of advanced-stage disease and its relapse.

An emerging field of investigation is using ctDNA for cancer screening and early detection to identify the carcinogenesis process before the development of invasive cancer. However, the biggest challenge is the sensitivity because of the low concentration of ctDNA in the blood, so this clinical application remained unsolved.

In considering the future clinical implementation of liquid biopsies, several studies have suggested that ctDNA can be prognostic in assessing MRD in different cancer subtypes, including lung cancer. The possibility of detecting MRD after curative-intent treatment and before radiological imaging remains a challenging opportunity to select patients who could really benefit from adjuvant therapies.

In the past few years, the treatment of metastatic NSCLC evolved from a non-specific cytotoxic chemotherapy to a personalized treatment based on genotype-directed therapy. In the current era of precision medicine, international guidelines recommended a broad-based analysis by NGS for molecular profiling. Unfortunately, tumor biopsy samples are not always adequate for molecular testing. Liquid biopsy has, therefore, emerged as a promising approach to detect genetic alterations in ctDNA without tissue biopsy risks and specimen limitations. Currently used as a complementary tool to the tissue biopsy in metastatic setting, liquid biopsy has gained much attention as a powerful technique to monitor real-time tumor dynamics. It is well-known that the emergence of resistance alterations inevitably limits the efficacy of targeted therapies. Many studies have shown the molecular tumor heterogeneity and how the cancer genomic profile may change over time. Liquid biopsy, allowing for serial assessments over time, has the potential to overcome tumor heterogeneity and permits longitudinal treatment response, detecting and quantifying the molecular alterations and also individualizing tumor subclones that harbour resistance mechanisms before clinical and imaging evaluations. Obviously, this implies an increasing complexity of the available information in terms of molecular alterations, mandating the presence of dedicated multidisciplinary molecular tumor boards capable of interpreting and providing indications with a useful clinical impact.

The future implementation of liquid biopsy in clinical practice may eventually permit a plasma-first treatment approach for oncogene-driven NSCLC, where plasma biomarker determines the treatment choice, overcoming tissue sampling. These also include differentiating and predicting immune checkpoint blockade response patterns.

As highlighted in our review, liquid biopsy has emerged as a new promising biomarker both in early and advanced NSCLC. We have reported that, although the many studies and advances in the last few years concerning the meaningful utility of liquid biopsy, little of its potential applications have been translated into clinical practice due to several limitations correlated to liquid biopsy. Indeed, despite all the progress made in blood-based technology, this approach still lacks standardization and analyte validation in clinical trials to assess accuracy, sensitivity, specificity, and predictive values.

Although accuracy in tumor profile precision has improved, the risk for false-positive or false-negative results remains. Therefore, the FDA has recommended a tissue biopsy to confirm a negative result from a liquid biopsy test with Guardant360 CDx and FoundationOne Liquid CDx. The National Comprehensive Cancer Network (NCCN) Clinical Practice Guidelines specify that ctDNA testing should not replace a histological tissue diagnosis, but it can be considered for molecular analysis of advanced NSCLC patients medically unfit for invasive tissue biopsy [198].

The International Association for the Study of Lung Cancer (IASLC) has provided a recent consensus statement on the use of liquid biopsy in advanced NSCLC, declaring that blood-based analysis in oncogene-addicted NSCLC patients is complementary to tissue-based analysis [6].

Therefore, recent advances in the characterization of ctDNA have demonstrated that liquid biopsy and tumor tissue analysis could be complementary, rather than alternative, techniques for molecular profiling in advanced NSCLC.

In conclusion, while ctDNA analysis is widely used in clinical practice for molecular profiling in advanced NSCLC, the additional clinical opportunities for treatment monitoring, MRD evaluation, and early cancer detection remain to be proven. Therefore, ctDNA assays should not be used outside translational research for these applications.

The improvement of liquid biopsy techniques, both to isolate analytes and to analyze them, is required to increase the sensitivities and specificities of the tests.

In the last years, with more and more new tiles provided, the potential key role of liquid biopsy has been enhanced as a game-changer in clinal practice. In the near future, we hope that the continued implementation of liquid biopsy testing, and the development of large clinical validation trials, will confirm and increase the potential of the clinical role of liquid biopsy in lung cancer management, improving patient outcomes by enabling early cancer detection, MRD assessment, and real-time monitoring of tumor treatments.

## Figures and Tables

**Table 1:** ctDNA performance to detect MRD in early-stage NSCLC.

Study	Number of Patients	Clinical Stage	ctDNA Methodology	Mutations Monitored	Treatment	ctDNA MRD Landmark	
						Sensitivity	Specificity
Chaudhuri et al. [103]	37	IB–IIIB	CAPP-Seq	Multiple	CRT or RT and/or surgery ± chemotherapy	94%	100%
Chen et al. [74]	25	IIB–IIIB	cSMART	Multiple	Surgery ± chemotherapy	44%	88%
Moding et al. [104]	12	IIB–IIIB	CAPP-Seq	Multiple	CRT	100%	100%
Abbosh et al. [30]	108	IA–III	Anchored-multiplex PCR (AMP)	Multiple	Surgery ± chemotherapy	49%	N.E. *

Abbreviations: ctDNA, circulating tumor DNA; MRD, minimal residual disease; NSCLC, non–small-cell lung cancer; CAPP-Seq, Cancer Personalized Profiling by deep Sequencing; CRT, chemoradiation; cSMART, circulating singlemolecule amplification and resequencing technology; PORT, postoperative radiotherapy; RT, radiotherapy. Clinical sensitivity (percentage of patients who relapsed in the follow up period who were ctDNA positive) and clinical specificity (percentage of patients who did not relapse in the follow up period who were ctDNA negative) were calculated for the first follow up sample after completing definitive therapy (ctDNA MRD Landmark). * In the paper, a positive predictive value of landmark has been reported for relapse of 93% and a negative predictive value of landmark for relapse of 68%.

**Table 2:** ctDNA plasma detection of EGFR mutations in liquid biopsy from advanced NSCLC.

Study	Sample Size	Method of Detection	Type of Sample	Alteration Detected	Sensitivity
Douillard et al. [52]	652	QUIAGEN therascreen EGFR RGQ PCR Kit	cfDNA (plasma)	EGFR-sensitizing mutations	65.7%
Reck et al. [49]	1162	QUIAGEN therascreen EGFR RGQ PCR Kit; Cobas EGFR mutations test version 2; Cycleave; PNA-LNA PCR Clamp; other	ctDNA	EGFR-sensitizing mutations	46%
Wu et al. [110]	334 (plasma) 287 (serum)	QUIAGEN therascreen EGFR RGQ PCR Kit	cfDNA	EGFR-sensitizing mutations	60.5% (plasma) 28.6% (serum)
Han et al. [111]	2561	Cobas EGFR mutations test version 2	ctDNA	EGFR-sensitizing mutations	46.9%
Karlovich et al. [112]	153	Cobas EGFR mutations test version 2; BEAMing (Symex Inostics GmbH)	cfDNA (plasma)	EGFR-sensitizing mutations and T790M resistance mutation	Activating mutations: 73%/82% T790M: 64%/73%
Oxnard et al. [114]	216	BEAMing (Sysmex Inostics GmbH)	cfDNA (plasma)	EGFR-sensitizing mutations and T790M resistance mutation	T790M: 70% L858R: 86% Del 19: 82%
Sacher et al. [113]	180	Droplet digital PCR (ddPCR)	cfDNA (plasma)	EGFR-sensitizing mutations and T790M resistance mutation	L858R: 74% Del 19: 82% T790M: 77
Zheng et al. [115]	117	Droplet digital PCR (ddPCR)	ctDNA	T790M resistance mutation	81%

**Table 3:** Selection of studies evaluating ctDNA as predictive factor of response to ICIs.

Study	Number of Patients/Study Type	Treatment	Outcomes
Cabel et al. [182]	15 (NSCLC and other cancer types) patients/prospective, pilot study	≥2nd line nivolumab or pembrolizumab	ctDNA clearance after 8 weeks associated with improved OS and PFS
Goldberg et al. [180]	28 Stage IV NSCLC patients/prospective	Anti-PD-L1/PD-1 ± anti-CTLA4	Reduction in ctDNA levels <50% from baseline correlated with improved PFS, OS and better ORR
Van der Leest et al. [185]	100 Stage IV NSCLC patients/retrospective	Anti-PD-L1/PD-1, ≥1st line	ctDNA decrease at 4–6 weeks associated with improved PFS and OS
Thompson et al. [186]	45 Stage IV NSCLC patients/prospective	Pembrolizumab ± chemotherapy, ≥1st line	A decrease >50% of ctDNA at 3 weeks correlated with better ORR, PFS and OS
Ricciuti et al. [181]	45 Stage IV NSCLC patients/retrospective	Pembrolizumab ± chemotherapy, 1st line	Decrease of ctDNA at 3 weeks associated with improved PFS, OS and better ORR
Ren et al. [188]	134 Stage III and IV NSCLC patients/exploratory analysis of phase III trial	Camrelizumab + chemotherapy, 1st line	ctDNA clearance or decrease after two cycles correlated with improved PFS and OS
Mondelo-Macia et al. [189]	50 Stage advanced NSCLC/prospective	Pembrolizumab ± chemotherapy, 1st line	Decrease of ctDNA at 12 weeks associated with improved PFS
Anagnostou et al. [190]	24 Stage IV NSCLC/retrospective	Anti-PD-1 ± anti-CTLA4/antiLAG3/chemotherapy, ≥1st line	ctDNA clearance associated with improved PFS and OS
Bratman et al. [187]	106 Stage IV patients (NSCLC and other cancer types)	Pembrolizumab, ≥1st line	ctDNA clearance associated with improved PFS and OS

## Data Availability

Not applicable.
